# Identification of a novel secreted metabolite cyclo(phenylalanyl-prolyl) from *Batrachochytrium dendrobatidis* and its effect on *Galleria mellonella*

**DOI:** 10.1186/s12866-022-02680-1

**Published:** 2022-12-08

**Authors:** Amanda M. Starr, Masoud Zabet-Moghaddam, Michael San Francisco

**Affiliations:** 1 Bryant & Stratton College, 8141 Hull Street Road, Richmond, VA 23235 USA; 2 Department of Biological Sciences, Texas Tech University, Lubbock, TX 79409-3131 USA; 3 Absci, 18105 SE Mill Plain Blvd, Vancouver, WA 98683 USA

**Keywords:** Fungi, Secondary metabolite, Wax moth larvae, Mass spectrometry

## Abstract

**Background:**

The fungus, *Batrachochytrium dendrobatidis,* is the causative agent of chytridiomycosis and a leading cause of global decline in amphibian populations*.* The first stages of chytridiomycosis include: inflammation, hyperkeratosis, lethargy, loss of righting reflex, and disruption of internal electrolyte levels leading to eventual death of the host. Previous work indicates that *B. dendrobatidis* can produce immunomodulatory compounds and other secreted molecules that regulate the growth of the fungus. In this study, filtrates of the fungus grown in media and water were subjected to ultra-performance liquid chromatography-mass spectrometry and analyzed using Compound Discoverer 3.0.

**Results:**

Identification of cyclo(phenylalanyl-prolyl), chitobiose, and S-adenosylmethionine were verified by their retention times and fragmentation patterns from *B. dendrobatidis* supernatants. Previous studies have analyzed the effects of *B. dendrobatidis* on amphibian models, in vitro, or in cell culture. We studied the effects of live *B. dendrobatidis* cells, spent culture filtrates containing secreted metabolites, and cyclo(pheylalanyl-prolyl) on wax moth larvae (*Galleria mellonella)*. Concentrated filtrates caused melanization within 24 h, while live *B. dendrobatidis* caused melanization within 48 h.

**Conclusions:**

Here we show *B. dendrobatidis* produces secreted metabolites previously unreported. The impacts of these chemicals were tested on an alternate non-amphibian model system that has been used for other fungi to study pathogenicity traits in this fungus.

**Supplementary Information:**

The online version contains supplementary material available at 10.1186/s12866-022-02680-1.

## Background

Amphibians, including frogs, toads, salamanders, newts, and caecilians, have been declining at alarming rates since the 1970s [1]. A major contributor to global amphibian decline in the 1990s was determined to be the chytrid fungus *Batrachochytrium dendrobatidis (B. dendrobatidis)*, and in 2010 a decline in salamanders was attributed to *Batrachochytrium salamandrivorans (B. salamandrivorans)* [2–4]. *Batrachochytrium* species are currently known to infect between 50–55% of frogs, toads, salamanders, and newts globally and 29% of caecilian species [5]. *B. dendrobatidis* exists in two life stages, the motile zoospore and the sessile, reproductive zoosporangium. The motile zoospore can produce a germ tube and enzymes to degrade intracellular junctions, skin components, and amphibian anti-microbial peptides [6–8]. The zoospore matures into a zoosporangium, which is known to produce metabolites to inhibit B and T cell proliferation [9–11]. In vitro the zoosporangium also produces tryptophol, a quorum sensing compound, that is associated with aggregated growth [12]. Infected amphibians have symptoms of hyperkeratosis and slough skin to reduce infection loads. Severely infected individuals present neurological symptoms including loss of righting reflex and succumb to cardiac arrest [13, 14]. The reason for loss of righting reflex is unknown.

Fungi are known to produce primary metabolites, essential for growth and reproduction, and secondary metabolites, compounds not essential for growth or reproduction, but instead used to aid in survival [15]. Secondary metabolites of fungal origin that play roles in colonization and establishment of infections have been shown to contribute to cell death and neurological symptoms, and possess anti-microbial, anti-nematode, and anti-protozoal activity [16–20]. In the context of amphibian infections, we currently understand that such molecules may play roles in quorum sensing, interference with immunological responses, and establishment in the host [9, 10, 12, 21]. Proteins responsible for secondary metabolite synthesis may be encoded in gene clusters within the genome [22]. These metabolites have been identified through a variety of methods, but most success has been accomplished through use of liquid chromatography in tandem with mass spectrometry techniques [23]. *B. dendrobatidis* may possess genes that encode proteins responsible for secondary metabolite production [24].

Parasitic fungi have been studied using a variety of “non-primary hosts”. Non-amphibian model systems that have been used to study *B. dendrobatidis* infections are *Xenopus laevis* amphibian skin and cell lines (i.e. splenocytes, leukocytes, and macrophages), nematodes *(Caenorhabditis elegans*), zebra fish, and daphnia [9, 25–29]. Another model system used to study virulence mechanisms of fungi are wax moth larvae (*Galleria mellonella*) [30, 31]. Here we show, for the first time, that *B. dendrobatidis* can produce cyclo phenylalanyl-prolyl (cPP) during in vitro cell culture and this molecule can cause melanization in *G. mellonella* in a manner similar to live *B. dendrobatidis.* The selection of *G. mellonella* over a previously identified non-amphibian host was to explore the use of a different animal model not previously used with *B. dendrobatidis*.

## Results

### Identification of unique secreted metabolites produced by *Batrachochytrium dendrobatidis* in vitro

**Fig. 1:**
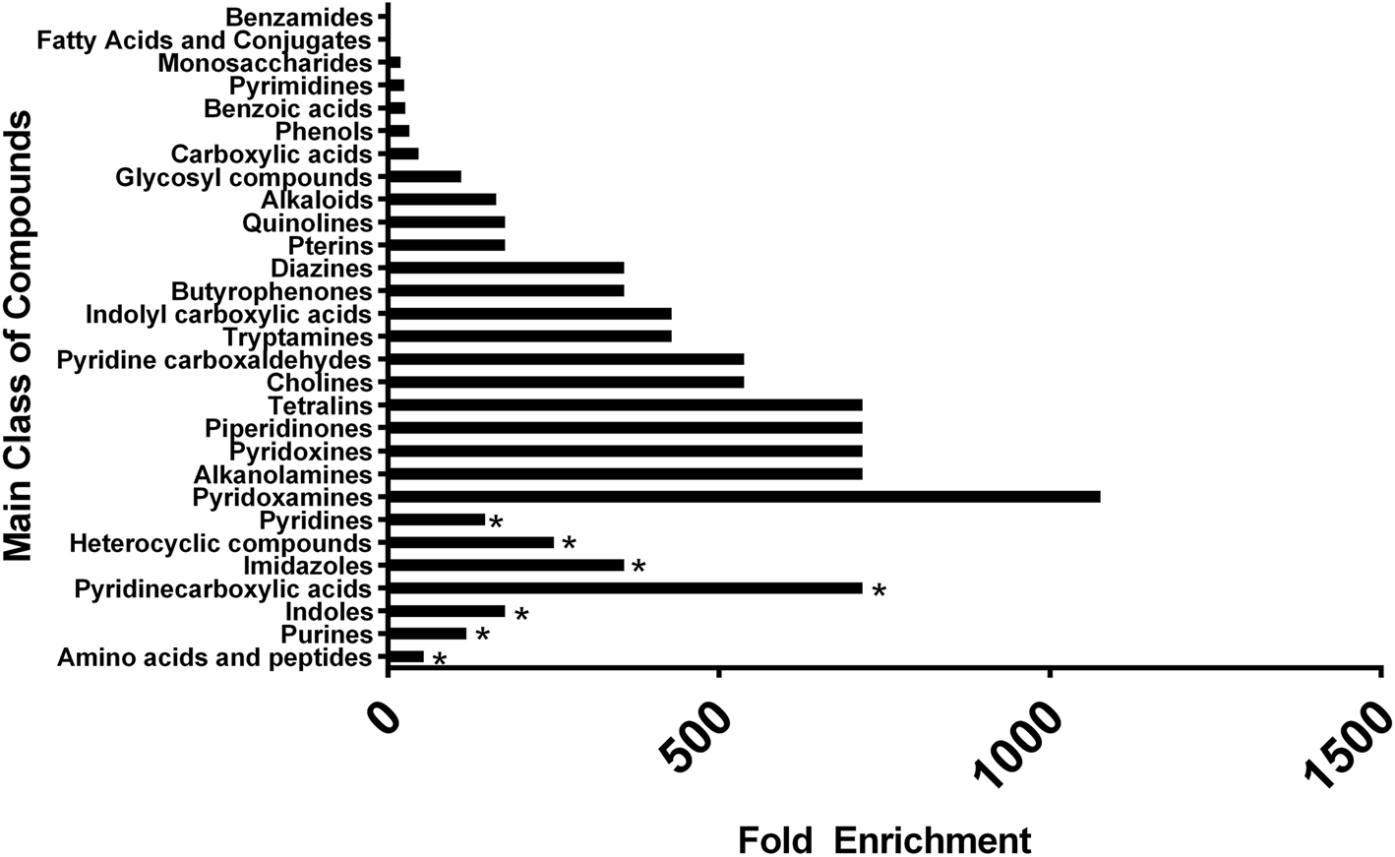
Over-representation analysis utilizing Metaboanalyst 5.1 of 92 metabolites secreted from *Batrachochytrium dendrobatidis* from water-*B. dendrobatidis* as compared to water (*N* = 3). Metabolite chemical structures were categorized by main class of compound and based on fold enrichment (number of hits/expected). * indicates those classes of compounds with enrichment values *p* < 0.05 and FDR < 0.05

**Fig. 2:**
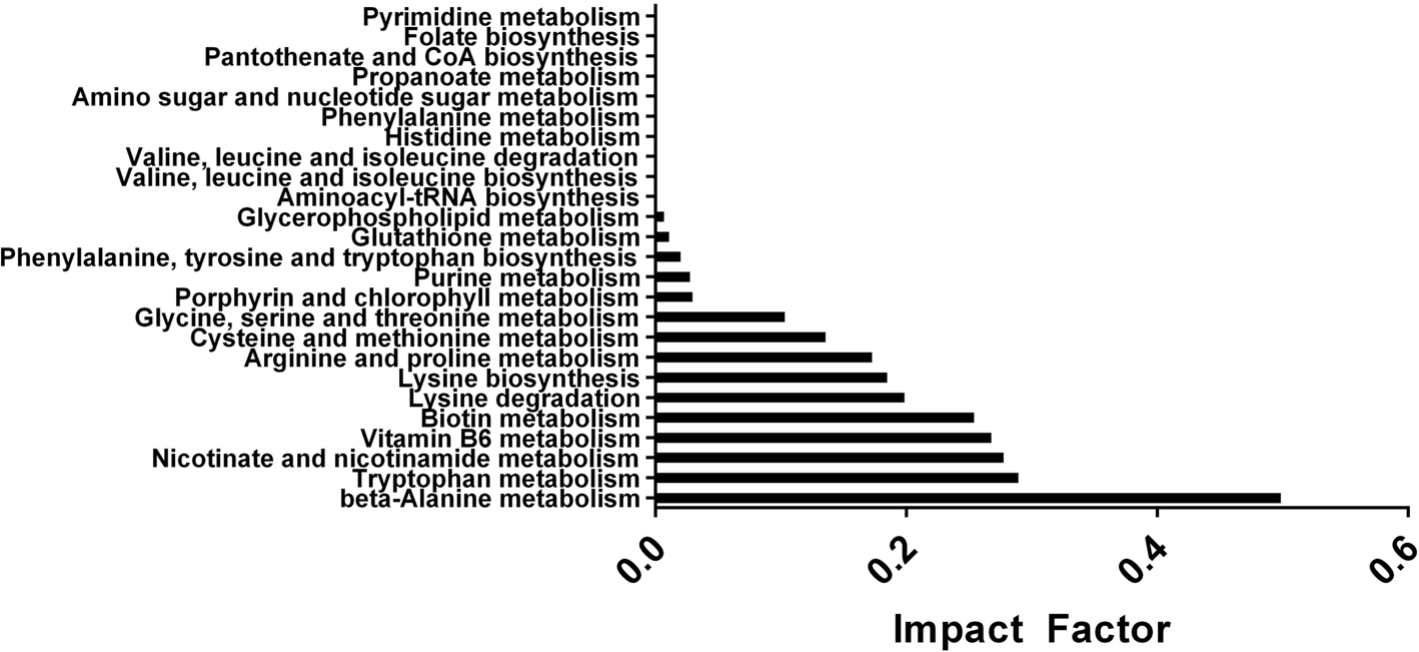
Metabolite pathway analysis utilizing Metaboanalyst 5.1 of 92 metabolites secreted from *Batrachochytrium dendrobatidis* from water-*B. dendrobatidis* as compared to water. *Saccharomyces cerevisiae* KEGG pathway used a base for analysis and based on impact factor (# metabolites from enrichment/centrality)

There were 2,285 compounds identified in the supernatant of *B. dendrobatidis* incubated in water compared to the media-water control. The list was narrowed by selecting compounds that had MS2 data, had an exact compound name, had a match to the mzCloud database, *p*-value < 0.01, and -2 < log_2_fold change > 2. Any repeat compound names were removed. There were 113 remaining compounds after the selection criteria (Sup Table 1). Four novel metabolites and previously known secreted metabolites from *B. dendrobatidis,* including methylthioadenosine, kynurenine, tryptophan, and tryptophol were observed in this study and are highlighted in Table 1. Using the enrichment metabolite set enrichment analysis (MSEA) module in Metaboanalyst 5.1, 92 of 113 compound names matched under the KEGG, PubChem, and Human Metabolome Database (HMDB). These 92 compounds were observed to be significantly enriched for amino acids and peptides, purines, indoles, pyridinecarboxylic acids, imidazoles, heterocyclic compounds, and pyridines (*p* < 0.05, FDR < 0.05) (Fig. 1). The pathway analysis module in Metaboanalyst 5.1 was also utilized with the following parameters: enrichment utilizing Fisher’s Exact Test, topology utilizing the relative-betweenness centrality, and the *Saccharomyces cerevisiae* KEGG pathway. The metabolic pathways these 92 compounds most affected were: beta-alanine, tryptophan, nicotinate/nicotinamide, vitamin B6, biotin, and lysine degradation (impact factor > 0.2) (Fig. 2).

**Table 1:** Identity of selected compounds secreted from water-*B. dendrobatidis* as compared to water. Known compounds produced from *B. dendrobatidis* verify results (*) and data showing the presence of unique compounds by water- *B. dendrobatidis* (**) (*N* = 3)

Compound Name	*Molecular Weight*	*RT (min)*	*Log*_*2*_*Fold*	*P-Value*
Unknown (QL or Similar to D-Carnitine)^**^	235.1415	0.826	15.26	2.23E-06
S-Adenosylmethionine^**^	398.1365	0.631	13.96	1.61E-05
5’-S-Methyl-5’-thioadenosine^*^	297.0891	0.599	12.35	9.27E-05
Chitobiose, di-N-acetyl^**^	424.1685	0.813	11.52	1.79E-06
Tryptophol^*^	161.0839	5.299	8.21	9.46E-05
Cyclo(phenylalanyl-prolyl) ^**^	244.1209	5.633	2.97	0.000926
DL-Tryptophan^*^	204.0897	3.312	2.88	0.039086
Cyclo(phenylalanyl-prolyl) ^**^	244.1209	5.481	2.87	0.000346
Kynurenine^*^	208.0846	3.043	2.38	0.003395

**Fig. 3:**
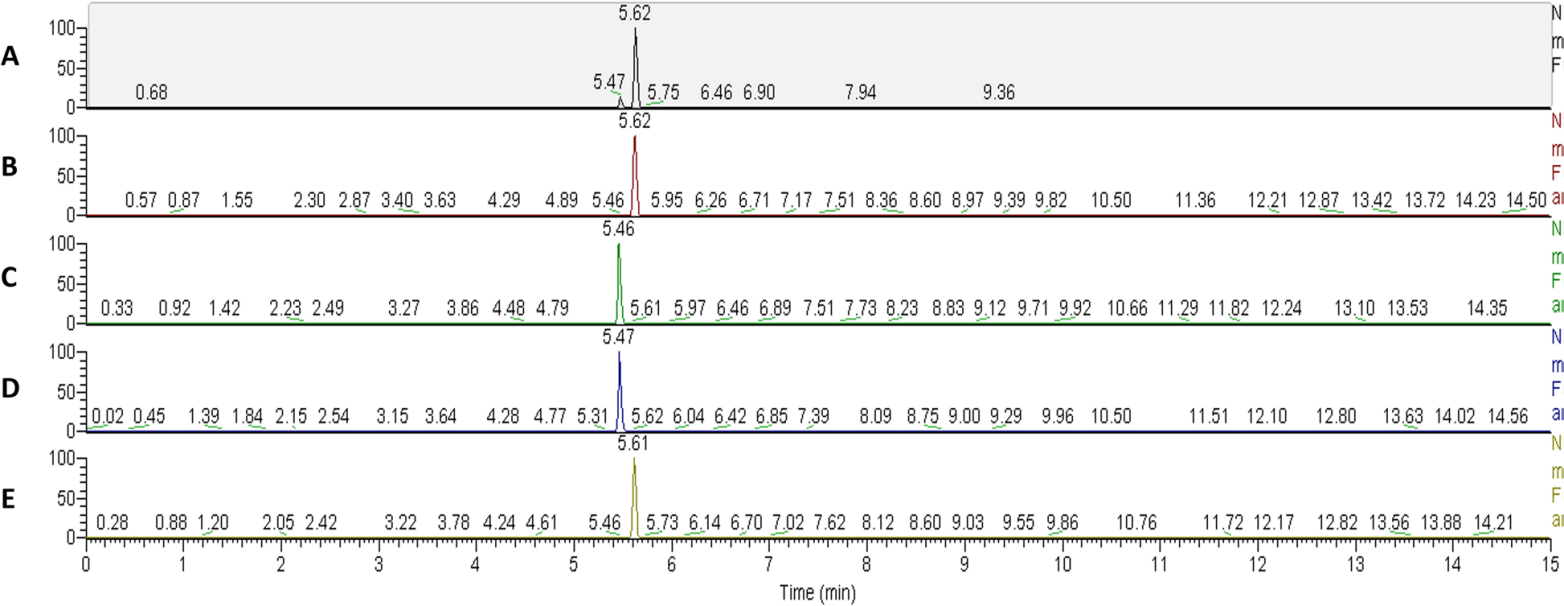
Ion chromatogram of LC-MS analysis of m/z 245.12 peak from **a**) *B. dendrobatidis* water, **b**) cyclo(L-Phe-L-Pro), **c**) cyclo(L-Phe-D-Pro), **d**) cyclo(D-Phe-L-Pro), and **e**) cyclo(D-Phe-D-Pro)

We detected unique peaks with m/z of 245.12, 399.14, 424.17, and 236.14. Further analysis with Compound Discoverer, identified the 245.12 peak as cyclo(phenylalanyl-prolyl) which was significantly increased in area under the peak in the *B. dendrobatidis*-water (*B. dendrobatidis* cultured in 50% H-broth with lactose (HBL) for 4 d, washed in water, and further incubated in water for 24 h) versus water (glass beads cultured in HBL for 4 d, washed in water, and further incubated in water for 24 h) (Log_2_Fold Change = 2.97, *P* < 0.000926). Standards of cyclo(L-Phe-L-Pro), cyclo(L-Phe-D-Pro), cyclo(D-Phe-L-Pro), and cyclo(D-Phe-D-Pro) were compared by mass spectrometry with those observed in the *B. dendrobatidis* sample at a concentration of 1 mM. The comparisons indicate that the smaller peak (5.4 min) is the cyclo(L-Phe-D-Pro) or cyclo(D-Phe-L-Pro) conformation, and the larger peak (5.6 min) is the cyclo(L-Phe-L-Pro) or cyclo(D-Phe-D-Pro) conformation (Fig. 3). The 236.12 peak showed a compound similar to isopropyl aminoethylmethyl phosphonite (QL compound) or possesses a D-carnitine side chain following analysis with Compound Discoverer 3.0. The fraction containing the 236.12 peak was collected 20 times with 20 µl injections and used for bioassays. The fraction containing the 424.16 peak was identified as chitobiose and the 399.14 peak as S-adenosylmethionine.

### Melanization occurs and cocoon development is lacking in *Galleria mellonella* after injections with *Batrachochytrium dendrobatidis* cells and culture supernatants

**Fig. 4:**
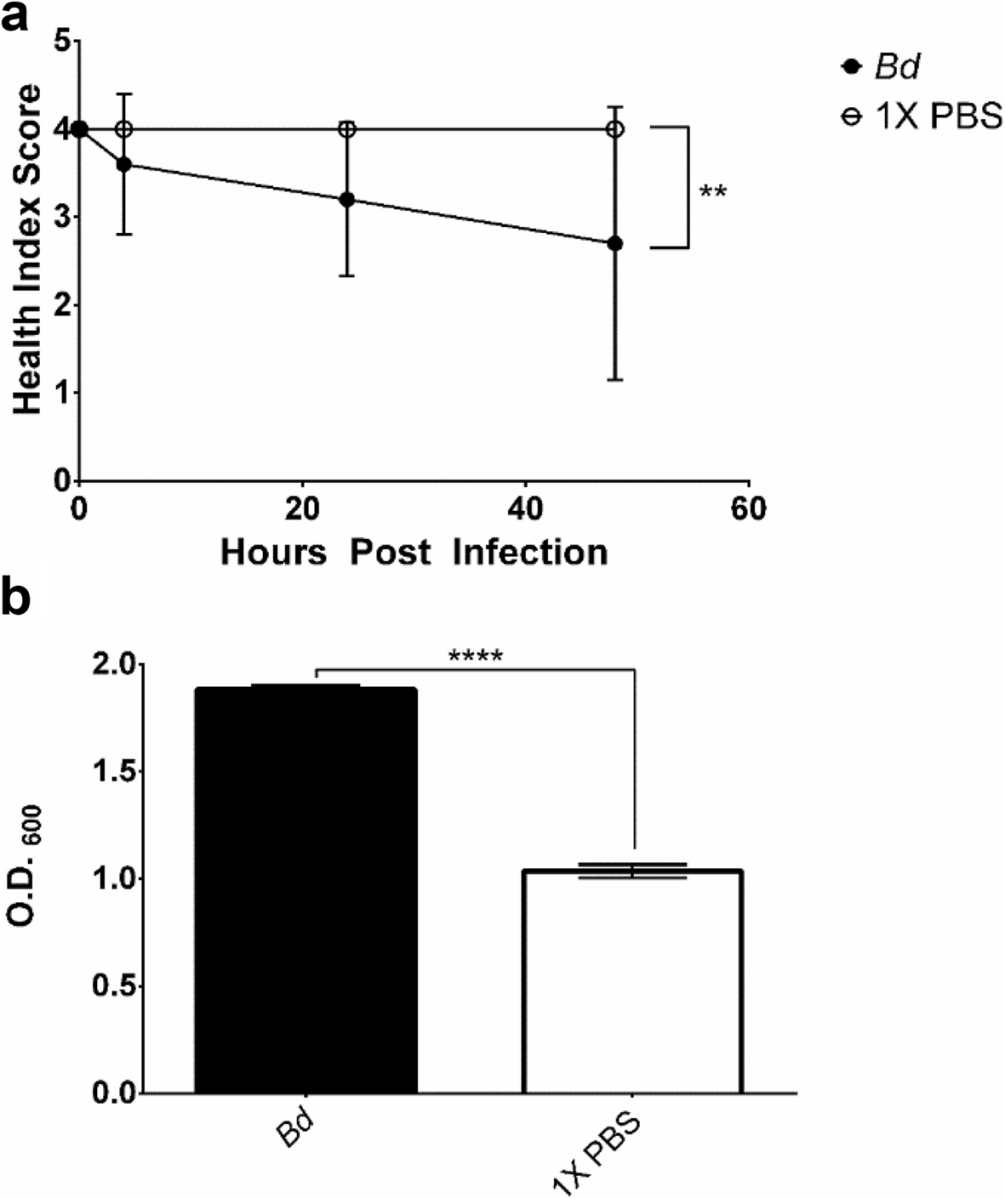
**A)** Health index score (degree of melanization) of wax moth larvae injected with *B. dendrobatidis* in 1X PBS and 1X PBS over 48 hrs of observations *N* = 10, ** *P* < 0.01, 3 replicates, and **B)** O.D. of hemocyte free hemolymph from larvae injected with *B. dendrobatidis* and 1X PBS, *N* = 10, 3 replicates, **** *P* < 0.0001

**Fig. 5:**
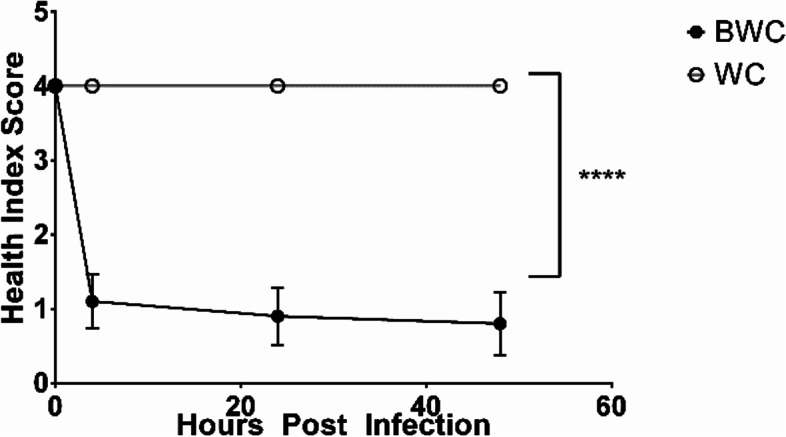
Health index score (degree of melanization) of wax moth larvae injected with *B. dendrobatidis* water 10X concentrated (BWC) and water control 10X concentrated (WC) over 48 hrs of observations *N* = 10, **** *P* < 0.0001, 3 replicates

**Fig. 6:**
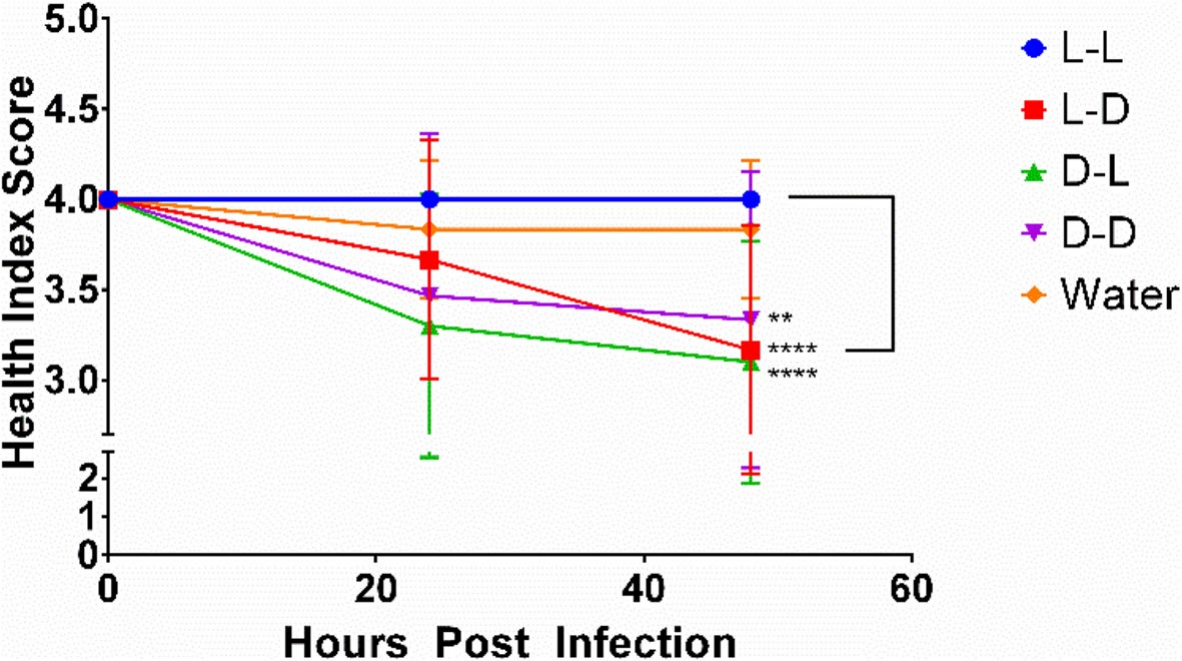
Health index score (degree of melanization) of wax moth larvae injected with 1 mM cyclo(L-Phe-L-Pro) (L-L), cyclo(L-Phe-D-Pro) (L-D), cyclo(D-Phe-L-Pro) (D-L), cyclo(D-Phe-D-Pro) (D-D), and control water (Water) over 48 hrs of observation. *N* = 10, **** *P* < 0.0001, **P < 0.01, 3 replicates

**Fig. 7:**
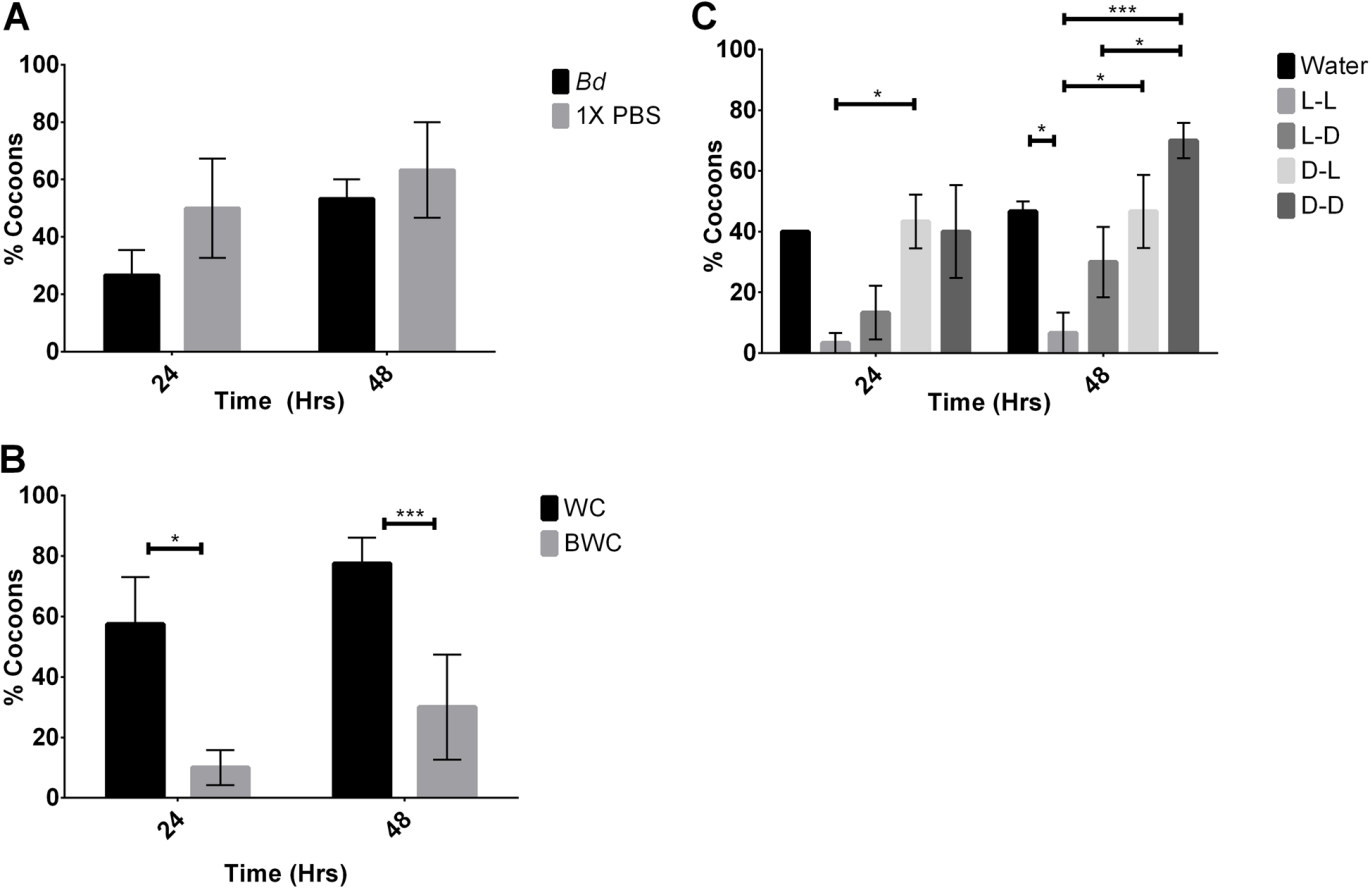
Cocoon development of wax moth larvae injected with: **A**) *B. dendrobatidis (Bd)* and 1X PBS over 48 hrs observation. *N* = 10, 3 replicates, **B**) *B. dendrobatidis* water 10X concentrated (BWC) and water control 10X (WC) over 48 hrs of observations. *N* = 10, ***P < 0.001, * *P* < 0.05, 3 replicates, **C)** 1 mM cyclo(L-Phe-L-Pro) (L-L), cyclo(L-Phe-D-Pro) (L-D), cyclo(D-Phe-L-Pro) (D-L), cyclo(D-Phe-D-Pro) (D-D), and control water (Water) over 48 hrs of observation. *N* = 10, *** *P* < 0.001, *P < 0.015, 3 replicates

The health score index based on melanization of wax moth larvae was significantly reduced when exposed to 10^7^ *B. dendrobatidis* cells (cultured in modified H-Broth 4 d, washed, and re-suspended in 1X PBS) after 48 hrs of exposure (Fig. 4a, *P* < 0.01) [32]. Analysis of hemocyte-free hemolymph showed significant melanization in the *B. dendrobatidis-* injected larvae compared to the 1X PBS injected larvae (Fig. 4b, *P* < 0.0001). A significant reduction of health score index was observed within 24 hrs after injection with concentrated water supernatant from *B. dendrobatidis* (Fig. 5, *P* < 0.0001). Use of pure cyclo(L-Phe-D-Pro), cyclo(D-Phe-L-Pro), and cyclo(D-Phe-D-Pro) conformations in our wax moth larvae model significantly decreased the health score index of the larvae within 48 hrs (Fig. 6, *P* < 0.0001, *P* < 0.0001, and *P* < 0.01, respectively). Injection of larvae with the fraction containing the 236 analyte (QL reagent or D-carnitine containing molecule) resulted in no significant melanization or cocoon development (Sup Fig. 1). Cocoon development was significantly decreased with the use of concentrated *B. dendrobatidis* supernatant in water and with injections of cyclo(L-Phe-L-Pro) and cyclo (L-Phe-D-Pro) but was not hindered with *B. dendrobatidis* cultures in PBS, PBS, concentrated water, cyclo(D-Phe-D-Pro), or cyclo(D-Phe-L-Pro) (Fig. 7, *P* < 0.05, *P* < 0.001).

## Discussion

Novel metabolites produced by *B. dendrobatidis* included a small cyclic dipeptide, cyclo(phenylalanyl-prolyl) (cPP), a QL compound related to carnitine, and numerous others. Identification of metabolites secreted by *B. dendrobatidis* could help with the development of vaccine targets for the prevention of infection and are essential to determine *B. dendrobatidis* impact on the environment and how the fungus can alter the ecology of amphibians [33]. Cyclo(phenylalanyl-prolyl) was also injected into a known model for studying fungal pathogenic traits, the waxmoth larvae, *G. mellonella.* The results showed total and partial melanization (identified by qualitative and quantitative means) which is one of the primary methods of determining toxicity. The addition of a non-vertebrate species for which *B. dendrobatidis* could be studied may allow for a more economical means for some researchers to study pathogenic traits.

The first metabolite focused on was the cyclo(phenylalanyl-prolyl) (cPP) a small cyclic dipeptide. While the cyclo(L-Phe-L-Pro) conformation of cPP is known to be produced by eukaryotes, we show *B. dendrobatidis* can potentially make all four conformations of the cyclic dipeptide (Fig. 3). Cyclic dipeptides are known to have anti-bacterial and anti-fungal activities, moderate gene expression, alter cell growth and apoptosis, interfere with toxin production, alter auxin related gene activity, and impact quorum sensing [34–41]. In addition, cyclic dipeptides are capable of inhibiting calcium channels [42]. Calcium channels are required for basic neurological responses, as calcium allows for the fusion of neurotransmitter membrane packets to enter neurons. We postulate calcium channels are inhibited during infection with *B. dendrobatidis*, which would inhibit the release of neurotransmitters in the amphibian. Furthermore, D-amino acids such as the cPP molecules identified in this study play a role in immune suppression and central nervous system health and disease [43]. This finding may explain why infected amphibians often experience the loss of the righting reflex.

The biosynthesis of cPP by non-ribosomal peptide synthetases (NRPS) is well-documented [15, 44–46]. *Bacillus brevis* is known to produce a cPP molecule, through a NRPS (*TycA)* [47]*.* The NRPS amino acid sequence in *B. brevis* was used as a probe for NRPS sequences in *B. dendrobatidis*. We found that *B. dendrobatidis* encodes proteins with high similarity to the *B. brevis* non-ribosomal peptide synthetase (20 potential matches, e value < 9E-06) (Table 2). While the cyclo(L-Phe-L-Pro) conformation of cPP is known to be produced by eukaryotes, we show *B. dendrobatidis* can potentially make all four conformations of the cyclic dipeptide (Fig. 3). The cyclo(L-Phe-D-Pro), cyclo(D-Phe-L-Pro), and cyclo(D-Phe-D-Pro) conformations of cPP produced a significant decrease in the health score index (significant increase in melanization of the larvae) in our wax moth larvae model system. Prophenoloxidase, an enzyme that is activated during the innate immune response of insects, causes melanization [48, 49]. This enzyme level is directly linked to the calcium concentrations in the hemolymph of the wax moth larvae. In high concentrations of calcium, this enzyme slows the activation of the prophenoloxidase system and prevents coagulation and healing to occur in the wax moth larvae [50, 51]. Our findings suggest that the presence of the D-amino acid in cPP plays a role in the induction of an immune response in the wax moth larvae, as evidenced by the production of melanization when the insects were exposed to the cyclo(L-Phe-D-Pro), cyclo(D-Phe-L-Pro), and cyclo(D-Phe-D-Pro) conformations. Additionally, the conformations that contain the D-isomers may contribute to a loss of the righting reflex seen in chytridiomycosis, since D-amino acids are largely found in neurotransmitters and the melanization was not inhibited during infection indicating lower calcium levels [52]. We interpret the above results to suggest that the cPP compound produced by *B. dendrobatidis* may aid in the loss of righting reflex seen in amphibians by inhibiting calcium channels and competing with receptors for neurotransmitters. Cyclic dipeptides with the cyclo (L-Phe-L-Pro), cyclo (L-Phe-D-Pro), and concentrates of *B. dendrobatidis* culture filtrates were also capable of inhibiting cocoon development in wax moth larva. Cyclic peptides are known to impact insect development as well [53, 54]. Arrangements of cPP that contain a D- configuration appear to result in melanization, while cPP with a L- configuration appear to result in defects of insect development. Since melanization and cocoon development are limited, other methods to explore to study the impact of *B. dendrobatidis* whole cells, supernatants, and cPPs would be to add on enzymatic assays, fungal cell viability, or proteomic and/or transcriptomic response of larvae to the infections.

**Table 2:** Comparison of tyrodicine encoding genes in *Bacillus brevis* compared to *Batrachochytrium dendrobatidis* (*Bd*) amino acid sequences to determine putative tyrodicine-like molecule-encoding genes using NCBI BLAST

Protein in *Bacillus brevis*	Encoded function	*Bd* match E-value	*Bd* Encoded function
TycA (WP_137029047.1)	Peptide Synthetase	2e-86	Peptide Synthetase
TycB (WP_137029049.1)	Peptide Synthetase	3e-95	Peptide Synthetase
TycC (WP_137029046.1)	Peptide Synthetase	6e-92	Peptide Synthetase
TycD (AAC45931.1)	ABC Transporter	9e-83	ABC Transporter
TycE (AAC45932.1)	ABC Transporter	3e-73	ABC Transporter
TycF (RAT94638.1)	Thioesterase	0.25	Ubiquitin-protein ligase

Since cPP is present in modified H-Broth, *B. dendrobatidis* and media-glass beads were heat-killed prior to 24 hrs water incubation. Samples were processed through mass spectrometry and there was a significant increase in cPP in the *B. dendrobatidis*-water sample compared to heat killed *B. dendrobatidis,* media-water, or heat-killed media-water (Table 3). Heat-killed *B. dendrobatidis* did not significantly change ± 1 log fold change compared to controls. These observations together with melanization by *B. dendrobatidis*-water and pure cPP, and no melanization observed with media-water, support our contention that cPP is being produced by *B. dendrobatidis* and is not solely an artifact from the media.

**Table 3:** Peak area comparisons of compound m/z peak 245.12 identified as cyclo(phenylalanyl-prolyl) utilizing Compound Discoverer 3.0 software to calculate the log2fold change differences between controls (Heated *B. dendrobatidis*, Heated Media Water, and Media Water) and sample of interest (*B. dendrobatidis* Water) and their respective *p*-values

Comparisons	Log_2_Fold Change	*P*-Value
*B. dendrobatidis* Water/ Heated *B. dendrobatidis*	3.21	7.26E-06
*B. dendrobatidis* Water /Media Water	1.82	0.00357
*B. dendrobatidis* Water /Heated Media Water	4.27	7.48E-07
Heated *B. dendrobatidis* Water/Media Water	-1.39	0.003829
*B. dendrobatidis* Water /Heated *B. dendrobatidis* Water	1.06	0.011177

Our unknown compound with a mass of 235.12 shows high similarity to molecules possessing a carnitine side chain. Carnitine in fungi is known to aid in the utilization of alternative carbon sources and to promote growth [55]. This compound could be used in conjunction with tryptophol to regulate growth. This compound also shows high similarity to isopropyl aminoethylmethyl phosphonite (QL compound) and procainamide. Isopropyl aminoethylmethyl phosphonite is a precursor molecule to a nerve agent known as VX (an organophosphorus compound that is an acetylcholinesterase inhibitor) [56]. Procainamide is a medication that is used to correct arrhythmias by blocking sodium channels on heart muscle [57]. The injection of the 235.12 compound into the wax moth larvae model system, did not show a significant decrease in health score index or negatively impact cocoon development.

In addition to the identified novel compounds secreted by *B. dendrobatidis,* 99 more compounds were identified. Of these 99 compounds, 92 compounds were assessed for their impacts on compound class and metabolic pathways. A large portion of these metabolites were enriched in amino acids and peptides, purines, indoles, pyridinecarboxylic acids, imidazole, heterocyclic compounds, and pyridines. These secondary compounds have been known to modulate the host immune system [58], to have antibacterial, analgesic, antipyretic, and anti-inflammatory properties [21, 59], and are linked to precursor molecules for kynurenine, tryptophan, tryptophol, spermidine/polyamines, methylthioadenosine (MTA) [10], activation for transition between zoospore to zoosporangium and germ tube formation, [60] increased virulence [10], and vitamin biosynthesis [21, 61].

## Conclusions

In this study, over 99 compounds were identified to be produced by *B. dendrobatidis.* One metabolite with four possible conformations of cyclo (Phe-Pro) dipeptide, were injected separately into a known non-vertebrate model, waxmoth larvae, to study fungal pathogenic traits. This dipeptide (cPP) caused melanization and negatively impacted cocoon development. We interpret the above results to suggest that the cPP compound produced by *B. dendrobatidis* may aid in exacerbating symptoms observed in infected amphibians. The use of *G. mellonella,* a well-defined model to study fungal pathogenesis, may serve as a non-amphibian host to explore pathogenesis by *B. dendrobatidis*.

## Materials and methods

### Growth conditions for the fungus

*B. dendrobatidis* VM1 isolate was obtained from a diseased Western Chorus Frog (*Pseudacris triseriata*) by Verma Miera and Elizabeth Davidson from Arizona State University and provided by Louise Rollins-Smith (Vanderbilt University). *B. dendrobatidis* was grown in 50% H-broth with lactose (HBL) (0.5% tryptone, 0.16% glucose, 2% lactose, w/v for all) with shaking in dark for 4 d until cell density of the culture grew to 10^7^ cells mL^−1^ as quantified by hemacytometer for all experiments. Media controls with HBL only and ~ 0.5 ml sterile 0.5 mm diameter glass beads were used to mimic volume of growing *B. dendrobatidis*. Cultures were centrifuged at 1,865 × g for 10 min. Cell culture pellets were washed twice with 2 mL HyClone Pure cell culture water. To determine if cPP was a media artifact, samples of HBL with glass beads and samples with living *B. dendrobatidis* cells were heated at 60 °C for 10 min. To confirm that heat treating killed *B. dendrobatidis* cells, 10 µl aliquots were grown on H-agar for 5 d. Heat-treated cells or beads were washed as described above. Tubes were shaken (0.03–0.1 × g) on a rotary shaker in the dark for 24 hrs. The cultures were centrifuged at 1,865 × g for 10 min and filtered with a 0.22 µm polyvinylidene difluoride (PDVF) filter to remove cells/glass beads. All samples were treated in triplicate.

### Ultra-Performance Liquid Chromatography-Mass Spectrometry (UPLC-MS/MS)

Five micro liter samples were collected from all conditions, *B. dendrobatidis* supernatant, cPP standards, and media and water controls, and analyzed by UPLCMS/ MS using Q Exactive™ HF Hybrid Quadrupole-Orbitrap™mass spectrometer (Thermo Scientific, USA). Standards of cyclo(L-Phe-L-Pro), cyclo(L-Phe-D-Pro), cyclo(D-Phe-L-Pro), and cyclo(D-Phe-D-Pro) were each dissolved at a concentration of 1 mM in water (Sigma-Aldrich, USA and Santa Cruz Biotechnology, USA). The chromatographic separation of *B. dendrobatidis* supernatant, cPP standards, and media and water controls were performed on Vanquish UPLC system using an Acquity UPLC column (2.1 × 150 mm, HSS T3 C18, 1.8 µm) (Thermo Scientific, USA). A 15-min gradient was used for separation using solvents A (water, 0.1% formic acid (FA) and B (methanol, 0.1% FA)). The gradient profile started with 0.5% solvent B, followed by an increase to 90% B in 9 min and then maintained at 90% B for another 1.5 min and finally decreased to 99.5% A for the equilibration of the column. The flow rate was kept at 0.45 µl min^−1^ during the run and column temperature was set at 55 °C throughout the run.

Data-dependent acquisition mode with two scan events was employed for MS/MS analysis. The first scan event was a full MS scan of 80–500 m*/z* at a mass resolution of 120,000. In the second scan event, the five most intense ions detected in the first scan event were selected to perform higher-energy C-trap dissociation (HCD) MS/MS with resolution of 15,000. An elevated normalized collision energy (NCE) from 20–60% was applied to obtain the MS/MS spectra. The dynamic exclusion was set to have repeat count of 2 and repeat duration of 6 s.

### Mass spectrometry data analysis

Metabolite structure search and predictions were done using Compound Discoverer 3.0 (Thermo Fisher Scientific, USA) based on molecular weight, retention time, and fragmentation pattern for each feature. Features from each data file were selected and aligned by retention time and molecular weight (mass tolerance of 5 ppm, intensity tolerance of 30%, min. peak intensity of 100,000). Common adducts of each feature were removed. Compound names were assigned to each feature with a similar match in the ChemSpider database or an elemental composition was assigned with Predict Compositions processing node (https://www.chemspider.com/Default.aspx). To identify more unknown features, mzLogic processing node compared MS2 data to the mzCloud database ( https://www.mzcloud.org/) for similar fragmentation. For those files with missing features, Fill Gap Nodes was applied to allow for statistical analysis. The identified compounds were then mapped to KEGG, Metabolika, and BioCyc Pathways.

Features were narrowed down to those that included ddMS2 data, had exact compound names, matched to the mzCloud database, had a *p*-value < 0.01, -2 < log_2_fold > 2, and no repeat compound names. Annotated feature intensities were normalized between *B. dendrobatidis*-water and media-water samples and area under the curve for each feature was compared using t-tests. Metaboanlayst 5.1 software was utilized for over-representation analysis and pathway analysis modules [62].

### Wax moth larvae bioassay

**Fig. 8:**
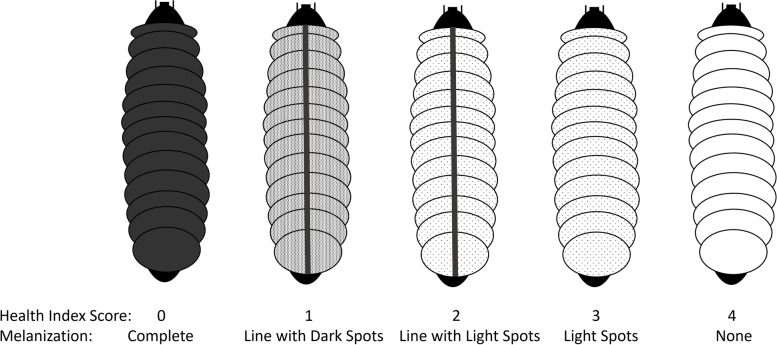
Visualization of the health index score to rank the level of melanization in wax moth larvae. A score of 4 is no melanization with a score of 0 being complete melanization and death of the larvae

The use of wax moth larvae, *Galleria mellonella*, is an accepted model to study microbial infections [30, 31]. Larvae were purchased from Carolina Biological (USA), allowed to rest 24 hrs in empty Petri dishes, and then treated as described below. All worms were used within 7 d of receipt. Supernatants or whole cells of *B. dendrobatidis* at a concentration of 10^7^ ml^−1^ in 1X PBS were used in these experiments. Solutions of cyclo(L-Phe-L-Pro) (Sigma-Aldrich, USA), cyclo(L-Phe-D-Pro), cyclo(D-Phe-L-Pro), and cyclo(D-Phe-D-Pro) (Santa Cruz Biotechnology, USA) at a concentration of 1 mM were dissolved in water for these experiments. In all cases larvae were injected into the left lower-proleg using sterile disposable syringes and sterile 25.5-gauge needles in 20 µl volumes into each of 10 worms in 3 independent replicates. Larvae not injected and those injected with only 1X PBS and water were used as controls. Worms were kept in sterile glass petri dishes and photographed every 24 hrs for 48 hrs and observed for the occurrence of melanization and cocoon development at 24 °C. After 2 d, worms were frozen for 4 d and autoclaved. Melanization characteristics of each larva were scored on a scale of 0–4 as previously described [63]. Photos of each group of larvae were scored blind based on melanization levels (health index score) to reduce bias (Fig. 8). The melanization curve was analyzed with a linear regression and two-way ANOVA with post-hoc Tukey’s analysis. The cocoon development was analyzed using two-way ANOVA with post-hoc Tukey’s analysis.

Thirty larvae were injected with *B. dendrobatidis* re-suspended in 1X PBS and 1X PBS control and incubated at 24 °C for 48 hrs. After incubation, the larvae were transferred to 4 °C for 15 min to immobilize and hemolymph extracted for analysis. Hemolymph was extracted by puncturing the lower abdomen with a sterile 25.5-gauge needle and outflowing hemolymph was collected in a 1.5 mL microcentrifuge tube. Hemolymph was centrifuged at 200 × g for 5 min at 4 °C to remove hemocytes, and the supernatant was centrifuged at 20,000 × g for 15 min at 4 °C to remove cellular debris. The degree of melanization of hemocyte-free hemolymph from each set of 10 injected larvae was measured as optical density (O.D.) at 600 nm. A Student’s t-test was performed to determine statistical significance.

## Supplementary Information

**Additional file 1:** **Sup Table 1.** Significant differential compounds secreted from water-*B. dendrobatidis* as compared to water identified by UPLC-MS/MS and Compound Discoverer 3.0. (*N*=3). **Sup Fig 1.** A) Health index score (degree of melanization) of wax moth larvae injected 236 analyte and 236 control over 48 hrs of observation. *N*=10, 3 replicates and B) Cocoon development of wax moth larvae injected with 236 analyte and 236 control over 48 hrs of observation. *N*=10, 3 replicates.

## Data Availability

The authors declare that data supporting the findings of this study are available within the article and it supplementary figures.

## Abbreviations

*G. mellonella*: *Galleria mellonella*
*B. dendrobatidis*: *Batrachochytrium dendrobatidis*
cP P: cyclo Phenylalanyl-prolyl
HBL 50%: H-broth with lactose
PDVF: Polyvinylidene difluoride
µm: Micrometers
min: Minutes
hrs: Hours
^o^C: Degree Celsius
x g: Relative centrifugal source
FA: Formic acid
HCD: Higher-energy C-trap dissociation
NCE: Normalized collision energy
UPLC-MS/MS: Ultra-Performance Liquid Chromatography-Mass Spectrometry
PBS: Phosphate buffered saline
O.D.: Optical density
HMDB: Human Metabolome Database

## References

[1] Wake DB, Vredenburg VT (2008). Are we in the midst of the sixth mass extinction? a view from the world of amphibians. Proc Natl Acad Sci.

[2] Longcore JE, Pessier AP, Nichols DK (1999). Batrachochytrium dendrobatidis gen. et sp. nov., a chytrid pathogenic to amphibians. Mycologia.

[3] Fisher MC, Garner TW (2020). Chytrid fungi and global amphibian declines. Nat Rev Microbiol.

[4] Martel A (2013). Batrachochytrium salamandrivorans sp. nov. causes lethal chytridiomycosis in amphibians. Proc Natl Acad Sci.

[5] Olson DH, et al. Global patterns of the fungal pathogen Batrachochytrium dendrobatidis support conservation urgency. Front Vet Sci. 2021. 8. 10.3389/fvets.2021.685877 PMC8322974 34336978

[6] Brutyn M (2012). Batrachochytrium dendrobatidis zoospore secretions rapidly disturb intercellular junctions in frog skin. Fungal Genet Biol.

[7] Moss A, Carty N, San Francisco M (2010). Identification and partial characterization of an elastolytic protease in the amphibian pathogen Batrachochytrium dendrobatidis. Dis Aquat Organ.

[8] Thekkiniath JC (2013). A novel subtilisin-like serine protease of Batrachochytrium dendrobatidis is induced by thyroid hormone and degrades antimicrobial peptides. Fungal Biol.

[9] Fites JS (2013). The invasive chytrid fungus of amphibians paralyzes lymphocyte responses. Science.

[10] Rollins-Smith LA (2015). Immunomodulatory metabolites released by the frog-killing fungus Batrachochytrium dendrobatidis. Infect Immun.

[11] Van Rooij P (2012). Germ tube mediated invasion of Batrachochytrium dendrobatidis in amphibian skin is host dependent. PLoS ONE.

[12] Verbrugghe E (2018). Growth regulation in amphibian pathogenic chytrid fungi by the quorum sensing metabolite tryptophol. Front Microbiol.

[13] Berger L, Speare R, Skerratt LF (2005). Distribution of Batrachochytrium dendrobatidis and pathology in the skin of green tree frogs Litoria caerulea with severe chytridiomycosis. Dis Aquat Org.

[14] Marcum RD (2010). Effects of Batrachochytrium dendrobatidis infection on ion concentrations in the boreal toad Anaxyrus (Bufo) boreas boreas. Dis Aquat Org.

[15] Keller NP, Turner G, Bennett JW (2005). Fungal secondary metabolism—from biochemistry to genomics. Nat Rev Microbiol.

[16] M’Barek SB (2015). FPLC and liquid-chromatography mass spectrometry identify candidate necrosis-inducing proteins from culture filtrates of the fungal wheat pathogen Zymoseptoria tritici. Fungal Genet Biol.

[17] Sava V (2006). Acute neurotoxic effects of the fungal metabolite ochratoxin-A. Neurotoxicology.

[18] Rodrigues KF, Hesse M, Werner C (2000). Antimicrobial activities of secondary metabolites produced by endophytic fungi from Spondias mombin. J Basic Microbiol.

[19] Anke H (1995). Secondary metabolites with nematicidal and antimicrobial activity from nematophagous fungi and Ascomycetes. Can J Bot.

[20] Gonçalves VN (2015). Antibacterial, antifungal and antiprotozoal activities of fungal communities present in different substrates from Antarctica. Polar Biol.

[21] Rollins-Smith LA (2019). Metabolites involved in immune evasion by Batrachochytrium dendrobatidis include the polyamine spermidine. Infect Immun.

[22] Smith D (1990). Beta-lactam antibiotic biosynthetic genes have been conserved in clusters in prokaryotes and eukaryotes. EMBO J.

[23] Nielsen KF, Larsen TO (2015). The importance of mass spectrometric dereplication in fungal secondary metabolite analysis. Front Microbiol.

[24] Bushley KE, Turgeon BG (2010). Phylogenomics reveals subfamilies of fungal nonribosomal peptide synthetases and their evolutionary relationships. BMC Evol Biol.

[25] Searle CL (2011). Differential host susceptibility to Batrachochytrium dendrobatidis, an emerging amphibian pathogen. Conserv Biol.

[26] Brannelly LA (2015). Batrachochytrium dendrobatidis in natural and farmed Louisiana crayfish populations: prevalence and implications. Dis Aquat Org.

[27] McMahon TA (2013). Chytrid fungus Batrachochytrium dendrobatidis has nonamphibian hosts and releases chemicals that cause pathology in the absence of infection. Proc Natl Acad Sci.

[28] Shapard E, Moss A, San Francisco M (2012). Batrachochytrium dendrobatidis can infect and cause mortality in the nematode Caenorhabditis elegans. Mycopathologia.

[29] Liew N (2017). Chytrid fungus infection in zebrafish demonstrates that the pathogen can parasitize non-amphibian vertebrate hosts. Nat Commun.

[30] Fuchs BB (2010). Methods for using Galleria mellonella as a model host to study fungal pathogenesis. Virulence.

[31] Mukherjee K (2010). Galleria mellonella as a model system for studying Listeria pathogenesis. Appl Environ Microbiol.

[32] Tsai CJ-Y, Loh JMS, Proft T (2016). Galleria mellonella infection models for the study of bacterial diseases and for antimicrobial drug testing. Virulence.

[33] McMahon TA (2014). Amphibians acquire resistance to live and dead fungus overcoming fungal immunosuppression. Nature.

[34] Nishanth Kumar S (2014). Cyclo (d‐Tyr‐d‐Phe): a new antibacterial, anticancer, and antioxidant cyclic dipeptide from Bacillus sp. N strain associated with a rhabditid entomopathogenic nematode. J Pept Sci.

[35] Ström K (2002). Lactobacillus plantarum MiLAB 393 produces the antifungal cyclic dipeptides cyclo (L-Phe-L-Pro) and cyclo (L-Phe-trans-4-OH-L-Pro) and 3-phenyllactic acid. Appl Environ Microbiol.

[36] Bina XR, Bina JE (2010). The cyclic dipeptide cyclo (Phe-Pro) inhibits cholera toxin and toxin-coregulated pilus production in O1 El Tor Vibrio cholerae. J Bacteriol.

[37] Kim K (2015). Cyclo (Phe-Pro) produced by the human pathogen Vibrio vulnificus inhibits host innate immune responses through the NF-κB pathway. Infect Immun.

[38] Ortiz-Castro R (2011). Transkingdom signaling based on bacterial cyclodipeptides with auxin activity in plants. Proc Natl Acad Sci.

[39] Holden MT (1999). Quorum-sensing cross talk: isolation and chemical characterization of cyclic dipeptides from Pseudomonas aeruginosa and other gram-negative bacteria. Mol Microbiol.

[40] Park D-K (2006). Cyclo (Phe-Pro) modulates the expression of ompU in Vibrio spp. J Bacteriol.

[41] Brauns SC (2004). Selected cyclic dipeptides inhibit cancer cell growth and induce apoptosis in HT-29 colon cancer cells. Anticancer Res.

[42] Milne P (1998). Medicinal chemistry: The biological activity of selected cyclic dipeptides. J Pharm Pharmacol.

[43] Fuchs SA (2005). D-amino acids in the central nervous system in health and disease. Mol Genet Metab.

[44] Wang N, Cui C-B, Li C-W (2016). A new cyclic dipeptide penicimutide: the activated production of cyclic dipeptides by introduction of neomycin-resistance in the marine-derived fungus Penicillium purpurogenum G59. Arch Pharmacal Res.

[45] Medema MH (2011). antiSMASH: rapid identification, annotation and analysis of secondary metabolite biosynthesis gene clusters in bacterial and fungal genome sequences. Nucleic Acids Res.

[46] Brakhage AA (2013). Regulation of fungal secondary metabolism. Nat Rev Microbiol.

[47] Mootz HD, Marahiel MA (1997). The tyrocidine biosynthesis operon of Bacillus brevis: complete nucleotide sequence and biochemical characterization of functional internal adenylation domains. J Bacteriol.

[48] Yang J (2012). An insecticidal protein from Xenorhabdus budapestensis that results in prophenoloxidase activation in the wax moth, Galleria mellonella. J Invertebr Pathol.

[49] Ratcliffe NA, Leonard C, Rowley AF (1984). Prophenoloxidase activation: nonself recognition and cell cooperation in insect immunity. Science.

[50] Kopácek P, Weise C, Götz P (1995). The prophenoloxidase from the wax moth Galleria mellonella: purification and characterization of the proenzyme. Insect Biochem Mol Biol.

[51] Mak TW, Saunders ME. The immune response. Part I: Basic Immunology, 2006: p. 373–401.

[52] Kiriyama, Y. and H. Nochi, D-amino acids in the nervous and endocrine systems. Scientifica, 2016. 2016. 10.1155/2016/6494621 PMC5178360 28053803

[53] Altstein M (2000). Advances in the application of neuropeptides in insect control. Crop Prot.

[54] Barbeta BL (2008). Plant cyclotides disrupt epithelial cells in the midgut of lepidopteran larvae. Proc Natl Acad Sci.

[55] Zhou H, Lorenz MC (2008). Carnitine acetyltransferases are required for growth on non-fermentable carbon sources but not for pathogenesis in Candida albicans. Microbiology.

[56] Bajgar J (2004). Organophosphates/nerve agent poisoning: mechanism of action, diagnosis, prophylaxis, and treatment. Adv Clin Chem.

[57] Colatsky T, Follmer C (1990). Potassium channels as targets for antiarrhythmic drug action. Drug Dev Res.

[58] Loenen, W., S-adenosylmethionine: jack of all trades and master of everything? 2006, Portland Press Limited. 10.1042/BST20060330 16545107

[59] Christopher SA (2002). Methylthioadenosine phosphorylase, a gene frequently codeleted with p16cdkN2a/ARF, acts as a tumor suppressor in a breast cancer cell line. Can Res.

[60] LS Kishbaugh, T., Pyridines and Imidazopyridines with medicinal significance. Curr Topics Med Chem. 2016. 16(28): p. 3274–3302. 10.2174/1568026616666160506145141 27150370

[61] Verbrugghe E (2019). Growth regulation in amphibian pathogenic chytrid fungi by the quorum sensing metabolite tryptophol. Front Microbiol.

[62] Chong J, Wishart DS, Xia J. Using MetaboAnalyst 4.0 for comprehensive and integrative metabolomics data analysis. Curr Protoc Bioinforma. 2019. 68(1): p. e86. 10.1002/cpbi.86 31756036

[63] Champion OL, Titball RW, Bates S. Standardization of G. mellonella larvae to provide reliable and reproducible results in the study of fungal pathogens. J Fungi. 2018. 4(3): p. 108. 10.3390/jof4030108 PMC6162639 30200639

