# Supplementary material for: Identification of a novel secreted metabolite cyclo(phenylalanyl-prolyl) from Batrachochytrium dendrobatidis and its effect on Galleria mellonella
Source: BMC Microbiol. 2022 Dec 8;22:293. doi: 10.1186/s12866-022-02680-1 (PMC9730576; doi:10.1186/s12866-022-02680-1)
Supplement: Supplementary file 1 — Additional file 1: Sup Table 1. Significant differential compounds secreted from water-B. dendrobatidis as compared to water identified by UPLC-MS/MS and Compound Discoverer 3.0. (N=3). Sup Fig 1. A) Health index score (degree of melanization) of wax moth larvae injected 236 analyte and 236 control over 48 hrs of observation. N=10, 3 replicates and B) Cocoon development of wax moth larvae injected with 236 analyte and 236 control over 48 hrs of observation. N=10, 3 replicates. [file 12866_2022_2680_MOESM1_ESM.docx]

| **Sup Table 1.** Significant differential **c**ompounds secreted from water-*B. dendrobatidis* as compared to water identified by UPLC-MS/MS and Compound Discoverer 3.0. (N=3). | | |
| --- | --- | --- |
| Name | Log_2_Fold Change | Adj. P Value |
| 1-Methylguanine | 11.09 | 7.3355E-05 |
| 7-Methylguanosine | 10.93 | 1.56606E-05 |
| 4-Acetamidobenzoic acid | 10.9 | 8.2168E-05 |
| 1-Methyladenine | 10.7 | 1.74069E-05 |
| 5'-S-Methyl-5'-thioadenosine | 10.3 | 1.85533E-05 |
| S-Adenosylhomocysteine | 10.27 | 9.35469E-05 |
| 7-Methylguanine | 10.05 | 6.25036E-05 |
| Methyl 2-[(2-methoxy-2-oxoethyl)amino]acetate | 9.82 | 5.55075E-05 |
| Putrescine | 9.67 | 2.99314E-05 |
| L-Threonine | 9.56 | 0.001717379 |
| Paracetamol | 9.47 | 0.00669053 |
| Tentoxin | 9.46 | 0.000125844 |
| Cytosine | 9.43 | 0.003186201 |
| Kynurenic acid | 9.38 | 4.42016E-05 |
| Isoxanthopterin | 9.16 | 0.001656504 |
| Guanine | 9.01 | 0.000332445 |
| N-Acetyl-DL-serine | 9.01 | 0.001625971 |
| α-Methyl-DL-histidine | 8.98 | 0.000291043 |
| Nicotinic acid | 8.97 | 0.005888798 |
| 2,5-Dimethylpyrazine | 8.94 | 2.44427E-05 |
| N-Acetyl-L-tyrosine | 8.91 | 7.3355E-05 |
| 5-Aminolevulinic acid | 8.85 | 0.000291043 |
| Indole-3-lactic acid | 8.84 | 0.003080642 |
| Methionine | 8.79 | 0.000301504 |
| N-Acetyl-DL-tryptophan | 8.79 | 0.000589267 |
| Imidazoleacetic acid | 8.66 | 8.2168E-05 |
| 4-Formylaminoantipyrine | 8.63 | 0.002632713 |
| 6-Methoxyquinoline | 8.62 | 0.005191594 |
| 6-Hydroxynicotinic acid | 8.56 | 0.002533901 |
| Pyridoxine | 8.56 | 0.002410611 |
| α-Aspartylphenylalanine | 8.45 | 6.81173E-05 |
| D-Serine | 8.34 | 0.000244087 |
| Benzocaine | 8.27 | 0.000589733 |
| L(-)-Carnitine | 8.23 | 0.007666051 |
| 2'-O-Methyladenosine | 8.21 | 0.002572523 |
| Nicotinamide | 8.2 | 0.003013675 |
| THJ | 8.18 | 0.000839004 |
| Xanthurenic acid | 8.08 | 0.000626726 |
| N-Acetyl-α-D-glucosamine | 8.03 | 0.000115731 |
| 3-[3-(Trifluoromethyl)phenyl]-1-(1,3,4-trimethyl-1H-pyrazolo[3,4-b]pyridin-6-yl)-1H-pyrazol-5-amine | 7.88 | 6.14855E-05 |
| L-Ergothioneine | 7.86 | 0.001043402 |
| Indole-3-acetic acid | 7.81 | 5.96003E-05 |
| β-Alanine | 7.76 | 0.001580175 |
| 2'-Deoxyadenosine | 7.69 | 2.76417E-05 |
| N6-Acetyl-L-lysine | 7.67 | 0.000138409 |
| tert-Butyl N-[1-(aminocarbonyl)-3-methylbutyl]carbamate | 7.63 | 0.00014541 |
| 5-Nitro-2-(1,2,3,6-tetrahydropyridin-1-yl)pyridine | 7.61 | 0.008452756 |
| Sedanolide | 7.58 | 0.008072736 |
| Valylproline | 7.56 | 1.74069E-05 |
| Biotin | 7.54 | 0.000213454 |
| Biocytin | 7.46 | 0.000117645 |
| N-Acetyl-L-leucine | 7.46 | 0.000175704 |
| N6,N6,N6-Trimethyl-L-lysine | 7.4 | 0.000271767 |
| L-Phenylalanine | 7.21 | 0.001422769 |
| 7,8-Dihydrobiopterin | 6.85 | 0.000353096 |
| Adenine | 6.84 | 0.000779919 |
| 2-(2,3-Dihydro-1,4-benzodioxin-6-yl)imidazo[1,2-a]pyrimidine | 6.83 | 0.005144293 |
| 1-Methylnicotinamide | 6.82 | 0.001586108 |
| DL-Stachydrine | 6.47 | 0.004728332 |
| N-Acetylputrescine | 6.45 | 0.000301504 |
| Azobenzene | 6.41 | 0.00020826 |
| Leucine | 6.29 | 0.000866237 |
| 1,5-Isoquinolinediol | 6.26 | 0.000853442 |
| 2-{2-Oxo-2-[4-(1H-pyrrol-1-yl)piperidino]ethoxy}acetic acid | 6.26 | 0.000587263 |
| 1,3-Divinyl-2-imidazolidinone | 6.24 | 0.005902627 |
| Pyridoxal | 6.22 | 0.000233305 |
| Methylimidazoleacetic acid | 6.21 | 0.000523162 |
| 4-Indolecarbaldehyde | 6.11 | 0.000671021 |
| D-(+)-Proline | 6.06 | 9.97429E-05 |
| 3-(2-Hydroxyethyl)indole | 5.95 | 0.003186201 |
| Pipecolic acid | 5.94 | 0.000993864 |
| N-Methylcaprolactam | 5.86 | 0.002121137 |
| Glycylproline | 5.82 | 0.002871455 |
| L-Saccharopine | 5.79 | 0.000721148 |
| 1,3-Dimethyl-6-morpholino-1,2,3,4-tetrahydropyrimidine-2,4-dione | 5.68 | 0.001594769 |
| 2-Aminooctanedioic acid | 5.47 | 0.000202126 |
| D-Glucosamine | 5.45 | 0.002894702 |
| 5-Hydroxyindole-3-acetic acid | 5.35 | 0.006354925 |
| L-Kynurenine | 5.29 | 0.005364285 |
| Pilocarpine | 5.16 | 1.56606E-05 |
| L-Aspartic acid β-benzyl ester | 5.12 | 0.005477185 |
| Hypoxanthine | 5.04 | 0.00841655 |
| Maltol | 5.03 | 0.004713317 |
| 4-Guanidinobutyric acid | 5.01 | 0.002238457 |
| Ecgonine | 4.97 | 0.000537671 |
| Acetylarginine | 4.9 | 0.000161715 |
| Prolylleucine | 4.78 | 0.000104818 |
| Isoleucine | 4.67 | 0.000898661 |
| Prolinamide | 4.67 | 0.000287318 |
| Adenosine 3'5'-cyclic monophosphate | 4.59 | 0.004999477 |
| Nikethamide 1-oxide | 4.55 | 0.001588751 |
| L-Pyroglutamic acid | 4.48 | 0.003219214 |
| L-2-Aminoadipic acid | 4.19 | 0.005821469 |
| Crotonic acid | 4.18 | 0.003834164 |
| 10-HDA | 4.01 | 0.00769754 |
| Norharman | 3.96 | 0.009398986 |
| δ-Valerolactam | 3.63 | 2.3097E-05 |
| 8-Hydroxyquinoline | 3.42 | 0.003896618 |
| Fenspiride | 3.29 | 0.003256413 |
| Desthiobiotin | 2.86 | 0.00043349 |
| N-Acetylserotonin | 2.79 | 8.2168E-05 |
| Choline | 2.78 | 0.006991283 |
| Levetiracetam | 2.5 | 0.00027138 |
| 3-(1-hydroxyethyl)-2,3,6,7,8,8a-hexahydropyrrolo[1,2-a]pyrazine-1,4-dione | 2.44 | 0.00015035 |
| Cyclo(leucylprolyl) | 2.41 | 9.56903E-05 |
| Nadoxolol | 2.36 | 3.10209E-05 |
| Cyclo(phenylalanyl-prolyl) | 2.33 | 5.31981E-05 |
| Edaravone | 2.31 | 0.010015675 |
| Carbidopa | 2.18 | 0.001249407 |
| Pyridoxamine | 2.15 | 0.000656449 |
| Diethanolamine | -3.48 | 0.0041024 |
| 1-(2-Morpholinophenyl)dihydro-1H-pyrrole-2,5-dione | -4.25 | 0.001285907 |
| 2,6-Dimethyl-5-(4-methylphenoxy)pyrimidin-4-ol | -5.88 | 0.001114055 |


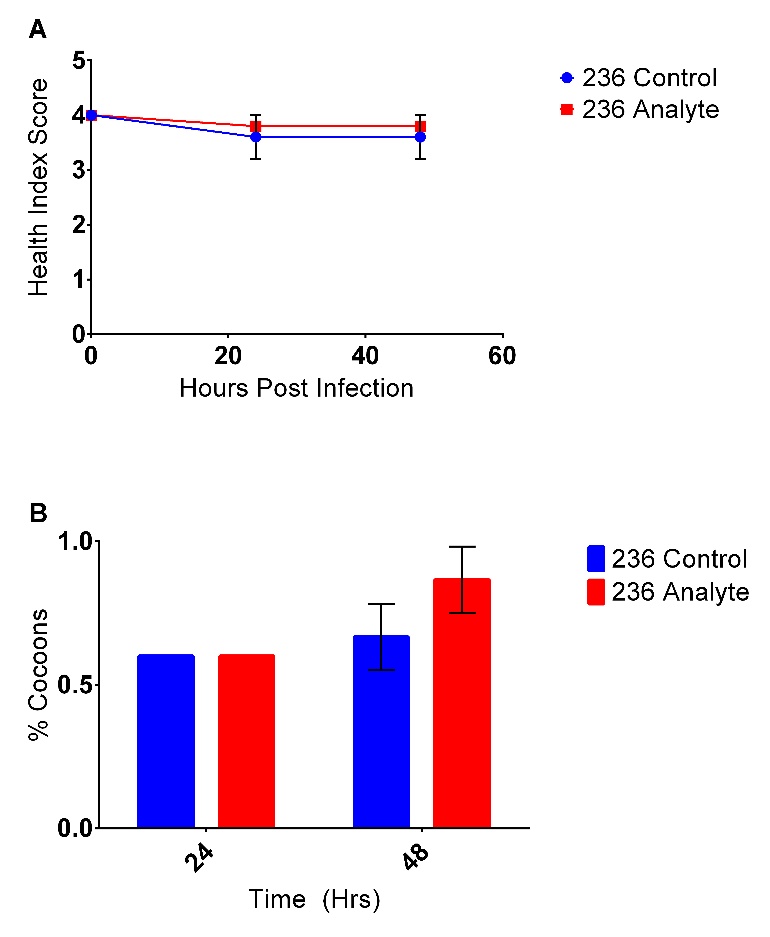


**Sup Fig 1** A) Health index score (degree of melanization) of wax moth larvae injected 236 analyte and 236 control over 48 hrs of observation. N=10, 3 replicates and B) Cocoon development of wax moth larvae injected with 236 analyte and 236 control over 48 hrs of observation. N=10, 3 replicates.
